# Awareness of fetal movements and care package to reduce fetal mortality (AFFIRM): a trial-based and model-based cost-effectiveness analysis from a stepped wedge, cluster-randomised trial

**DOI:** 10.1186/s12884-022-04563-9

**Published:** 2022-03-22

**Authors:** Elizabeth M. Camacho, Sonia Whyte, Sarah J. Stock, Christopher J. Weir, Jane E. Norman, Alexander E. P. Heazell

**Affiliations:** 1 Manchester Centre for Health Economics, School of Health Sciences, University of Manchester, Manchester, UK; 2 Liverpool Clinical Trials Centre, University of Liverpool, 1st Floor Block C, Waterhouse Building, 3 Brownlow Street, Liverpool, L69 3GL UK; 3 MRC Centre for Reproductive Health, University of Edinburgh, Edinburgh, UK; 4 Usher Institute, University of Edinburgh, Edinburgh, UK; 5 Faculty of Health Sciences, University of Bristol, Bristol, UK; 6 Maternal and Fetal Health Research Centre, School of Medical Sciences, University of Manchester, Manchester, UK

**Keywords:** Stillbirth, Perinatal death, Fetal movements, Cost-effectiveness, Randomised trial

## Abstract

**Background:**

The AFFIRM intervention aimed to reduce stillbirth and neonatal deaths by increasing awareness of reduced fetal movements (RFM) and implementing a care pathway when women present with RFM. Although there is uncertainty regarding the clinical effectiveness of the intervention, the aim of this analysis was to evaluate the cost-effectiveness.

**Methods:**

A stepped-wedge, cluster-randomised trial was conducted in thirty-three hospitals in the United Kingdom (UK) and Ireland. All women giving birth at the study sites during the analysis period were included in the study. The costs associated with implementing the intervention were estimated from audits of RFM attendances and electronic healthcare records. Trial data were used to estimate a cost per stillbirth prevented was for AFFIRM versus standard care. A decision analytic model was used to estimate the costs and number of perinatal deaths (stillbirths + early neonatal deaths) prevented if AFFIRM were rolled out across Great Britain for one year. Key assumptions were explored in sensitivity analyses.

**Results:**

Direct costs to implement AFFIRM were an estimated £95,126 per 1,000 births. Compared to standard care, the cost per stillbirth prevented was estimated to be between £86,478 and being dominated (higher costs, no benefit). The estimated healthcare budget impact of implementing AFFIRM across Great Britain was a cost increase of £61,851,400/year.

**Conclusions:**

Perinatal deaths are relatively rare events in the UK which can increase uncertainty in economic evaluations. This evaluation estimated a plausible range of costs to prevent baby deaths which can inform policy decisions in maternity services.

**Trial registration:**

The trial was registered with www.ClinicalTrials.gov, number NCT01777022.

**Supplementary Information:**

The online version contains supplementary material available at 10.1186/s12884-022-04563-9.

## Background

There are around 2,500 stillbirths per year in the UK [1], associated with an estimated annual cost to health and social services of £13.6 m in 2018 [2], with additional costs extending from the affected pregnancy into subsequent pregnancies [3]. The rate of stillbirths in the UK is higher than other countries in Western Europe and in 2015 ranked 24th highest out of 49 high-income countries [4]. A recently-published review of baby deaths and babies brain-damaged at birth in the UK reported that 74% of cases may have been avoidable [5]. Although there has been around a 10% reduction in the UK stillbirth rate since 2013 [6], the high proportion of avoidable deaths, coupled with the significant regional variation in stillbirth rates across the UK, suggests that there is scope to reduce it further [7].

Maternal perception of reduced fetal movements (RFM) is associated with increased likelihood of stillbirth [8, 9]. The AFFIRM intervention was designed to reduce stillbirth (and neonatal death) rates by increasing awareness of RFM among pregnant women and establishing a pathway of standardised management for women presenting with RFM [10]. A stepped-wedge cluster randomised trial was conducted to evaluate how effective AFFIRM was at reducing stillbirths [11]. The incidence of stillbirth was 4.40 per 1000 live births in the period before AFFIRM was implemented and 4.06 per 1000 live births afterwards (adjusted odds ratio 0.90, 95% CI 0.75–1.07). The change in the stillbirth rate observed was not statistically significant. This means that generally there were fewer stillbirths after AFFIRM was implemented, but there is a possibility that this was observed by chance.

Economic evaluations consider the likely costs and benefits of one intervention compared to an alternative. Unless there is strong evidence that the benefits of both alternatives are exactly equal, a comparison of costs and benefits is conducted in the form of a cost-effectiveness analysis, as this makes an important contribution to evidence-based decision-making in healthcare settings. A 2016 systematic review identified no cost-effectiveness evidence for interventions to enhance maternal awareness of RFM [12] and we have identified no subsequent relevant peer-reviewed publications. The economic evaluation reported here aimed to estimate the cost-effectiveness of the AFFIRM intervention compared to standard care, considering the uncertainty of the benefits associated with it. There are two parts to the economic evaluation; the first uses data from the AFFIRM trial to estimate the cost-effectiveness within the study sites (trial-based analysis), and the second uses a decision model to extrapolate data from the trial to estimate the costs and benefits of AFFIRM if it were rolled out across Great Britain for one year (model-based analysis).

## Methods

The full protocol for the AFFIRM trial has been published previously [10]. In brief, the study was a stepped wedge, cluster-randomised trial, whereby participating public maternity hospitals and maternity units in the UK and Ireland were randomised in clusters to one of nine intervention implementation dates. The clusters were hospitals, grouped geographically to minimise contamination, and each cluster had a total of around 17,000 births per year. Clusters were randomised using a computer-generated scheme, the implementation dates were concealed until 3 months before the implementation date. The time between each additional cluster moving into the intervention phase was 4 months. Data were collected for all births during the study period (January 2014 to December 2016), and only women who had asked to be withdrawn from routine data collection were excluded. Thirty-seven maternity units were randomised, but four withdrew from the study prior to implementing the intervention, leaving thirty-three units.

The AFFIRM intervention was a package of care which included promoting the importance of RFM awareness in pregnant women (a leaflet about RFM was given at around 20 weeks’ gestation) and healthcare professionals (through a bespoke e-learning education package), alongside a defined care plan for when women present with perceived RFM. The care plan included enhanced assessment of fetal wellbeing (ultrasound growth scans, liquor volume assessment, and cardiotocography) and, where the potential benefits were considered to outweigh any risks (e.g. ≥ 37 weeks gestation, recurrent RFM, presence of other risk factors), expedited delivery. The analysis compared outcomes in the period before the intervention was implemented (i.e. according to each maternity unit’s pre-AFFIRM standard operating procedures) versus outcomes following implementation. Births which occurred during the washout period (first 2 months after the implementation date) were not included in the analysis.

The analyses were conducted according to the intention-to-treat principle, i.e. regardless of the actual degree and timing of implementation of the intervention, but as per the planned implementation. All costs are reported in British pounds (£), and the price year was 2019. The UK NHS and personal social services perspective was used, in line with National Institute of Health and Care Excellence (NICE) guidance for economic evaluations of healthcare interventions [13]. STATA 15 (StataCorp. 2017. *Stata Statistical Software: Release 15*. College Station, TX: StataCorp LLC.) and TreeAge Pro Healthcare 2020 (TreeAge Pro 2020, R1. TreeAge Software, Williamstown, MA) were used to conduct the analysis. The trial was registered with www.ClinicalTrials.gov, number NCT01777022.

### Trial-based economic evaluation

The measure of health benefit for the trial-based economic evaluation was the difference in the number of (antepartum and intrapartum) stillbirths in the post-AFFIRM period compared to the pre-AFFIRM period, as reported in the published paper for the clinical effectiveness evaluation i.e. 5 fewer stillbirths per 10,000 births (95% CI 11 fewer to 3 more stillbirths) [11]. This was calculated using an approximate conversion from the odds ratios from the logistic regression models. In the original analysis, data regarding birth outcomes were derived from electronic healthcare records held in the National Safe Haven (NSH). As this study was based in the UK, the official UK definition of stillbirth was used, which is a baby born after 24 weeks which did not breathe or show signs of life [14]. Where gestation was uncertain, all babies with a birth weight of at least 500 g were assumed to be of 24 or more weeks’ gestation.

Costs included in the main analysis were: training of healthcare professionals in the AFFIRM intervention, leaflets given to pregnant women as part of the intervention, unplanned antenatal attendances due to RFM (in both treatment groups), and induction of labour (IoL). Costs for IoL were included as part of the package of care was to induce labour following presentation for RFM if there were concerns about the baby’s wellbeing. Sensitivity analyses included the costs of neonatal intensive care unit (NICU) admissions for > 48 h (this was a statistically significant outcome in the trial [11]), and intrapartum costs (vaginal birth or Caesarean-section delivery, excluding costs associated with IoL). Records of the number and role of all healthcare professionals who completed the 45-min online training program about the AFFIRM care package were made. Their roles were matched to NHS Agenda for Change salary bands to estimate a total training cost (described in more detail in Supplementary Material). A standardised audit proforma was provided for study sites to capture the number of RFM attendances and resulting additional ultrasound scans over a 28-day period. As audit data were not available for the whole study duration, it was assumed that the rate of attendances observed was constant over time. Data regarding IoLs, and NICU admissions were derived from electronic healthcare records. Unit costs were derived from the NHS reference costs database [15], the Personal Social Services Research Unit’s ‘Unit Costs of Health and Social Care’ [16], and original invoices (printing costs for the leaflets, inflated using health cost inflation indices [16]) – unit costs used in the analysis are reported in Supplementary Material (Table S1). The in-hospital costs following a stillbirth were derived from a UK population-based cost-of-illness study [2].

For the base-case (primary) economic analysis an incremental cost-effectiveness ratio (ICER) was calculated; accordingly, no parametric statistical tests of differences in mean costs or outcomes were conducted. The costs of pre-AFFIRM standard care and of post-AFFIRM care were estimated per 1,000 births. The incremental cost-effectiveness ratio was expressed as the cost per stillbirth avoided, calculated by dividing the difference in costs per 1000 births between the study periods by the difference in the stillbirth rate observed in the trial. One-way sensitivity analyses were conducted to explore the impact of design choices and analysis assumptions on the cost-effectiveness of AFFIRM. These were: impact of AFFIRM intervention on stillbirth rate, increase in RFM attendances, the proportion of RFM visits resulting in an additional ultrasound scan, and including/excluding costs for training, IoL, NICU admissions, mode of birth, and stillbirths (in-hospital costs).

### Model-based economic evaluation

The aim of the model-based analysis was to estimate the costs and benefits if AFFIRM were rolled out across the NHS in England, Wales, and Scotland (Great Britain). Therefore, the model cohort is the number of births in a one-year period in Great Britain. According to the Office for National Statistics (ONS), in 2019 there were 640,370 live births and 2,522 stillbirths in England and Wales [1] and according to the National Records of Scotland (NRS), in 2019 there were 49,863 live births and 174 stillbirths [17]. This gives a total cohort size of 692,929 births; a cohort of 693,000 was used in the model for simplicity.

There were some key differences between the trial- and model-based evaluations. The measure of health benefit in the trial-based analysis was the number of stillbirths. However, there was uncertainty associated with the impact of AFFIRM on the number of stillbirths. The sample size calculation conducted prior to the trial assumed a higher stillbirth rate than was observed during the trial. This meant that there was insufficient power to reliably detect whether there was a difference in the number of stillbirths before and after the AFFIRM care package was implemented. One way of trying to increase the power is to increase the number of “events” included in the analysis. For the model-based analysis, the number of perinatal deaths, which includes stillbirths *and* early neonatal deaths (within the first 7 days of life) was used as the measure of health benefit. Some babies were recorded as both stillbirths and early neonatal deaths in the trial database. In these cases, where an Apgar score > 0 or a NICU admission was recorded, it was assumed that the babies were born alive but subsequently died (i.e. that the baby was not stillborn). In addition, evaluating the number of perinatal deaths is argued to be the most appropriate outcome as this reflects the number of parents who had a live born baby that was still living at discharge from hospital; stillbirths and neonatal deaths are reported to have similar negative effects for parents [18]. A sensitivity analysis which included only stillbirths was also conducted. The trial analysis excluded babies who were stillborn at < 24 weeks’ gestation but included babies who were live born at fewer than 24 weeks’ gestation but died shortly after birth, counting them as live births. However, in the model-based analysis babies from either of these groups were excluded. Finally, for the trial-based analysis the mean level of resource use for the sample was used to estimate an overall cost associated with the intervention. But in the model-based analysis, each pathway of events (e.g. induction of labour vs. spontaneous; Caesarean section vs. vaginal birth etc.) had an associated cost based on the resources used, and the overall cost was estimated based on the probability of the different pathways occurring, which was derived from individual-level data.

A decision tree was constructed to represent the different birth outcomes (stillbirth, live birth with subsequent early neonatal death, live baby at 7 days postpartum—shown in Supplementary Material, Figure S1). The tree also depicts NICU admissions for babies who were live born, but these were not included in the primary analysis. The time horizon for the model was 7 days from birth.

The database used in the original AFFIRM analysis was used here to inform the model, so that proportionately the model cohort would follow the same pathways as was observed during AFFIRM. Logistic regression analysis was used to estimate the likelihood of the events in the decision tree. The confounders in the clinical effectiveness analysis were also adjusted for here. A random effect was included for randomisation cluster, and the intervention and study time periods (i.e. 4-monthly intervals when each additional cluster moved into the intervention phase) were fixed effects, maternal age and number of babies per pregnancy were also included as potential confounders. The probabilities used in the decision tree are reported in Supplementary Material (Table S2). Costs were included as per the trial-based economic evaluation and incorporate the intervention and events in the decision tree.

The costs, outcomes, and probabilities were entered into the model to estimate the cost-effectiveness of AFFIRM for the whole model cohort (i.e. the 693,000 births per year in Great Britain). One-way sensitivity analyses were conducted which were: including the estimated costs associated with NICU admissions, births, and baby deaths, and using stillbirths as the outcome measure. A probabilistic analysis was conducted whereby the value for each of the probabilities and costs in the primary (deterministic) model-based analysis were randomly selected from a distribution around the values. This generated a 95% confidence interval around the mean cost and mean outcome (i.e. number of perinatal deaths). A gamma distribution was used for costs (with α and λ derived from the individual unit costs and an estimated standard deviation of ± 20%) and a beta distribution for probabilities (with α and β derived from each probability and associated standard deviation reported in Supplementary Material (Table S2). A random seed of 383 (generated using random.org) was used.

## Results

The characteristics of the study population who gave birth at the participating maternity units are summarised in Table 1. There were no notable differences between the mothers or births in the pre- and post-AFFIRM study periods.

**Table 1:** Characteristics of mothers giving birth in the pre- and post-AFFIRM study periods (based on available data)

	Mean (SD) or *n*(%)	
	Control (pre-AFFIRM) *n* = 157,692	Intervention (post-AFFIRM) *n* = 227,860
Maternal age (years)	30.0 (5.8)	30.2 (5.7)
Ethnicity		
White Asian Black (African or Caribbean) Mixed Other Missing/not reported	118,127 (74.9%) 10,966 (7.0%) 4288 (2.7%) 2845 (1.8%) 2272 (1.4%) 19,194 (12.2%)	169,531 (74.5%) 15,144 (6.6%) 6172 (2.7%) 3221 (1.4%) 4126 (1.8%) 29,666 (13.0%)
BMI – overweight or obese (≥ 25 kg/m^2^)	61,950 (48.2%)	95,413 (49.9%)
Parity – nulliparous	65,145 (42.4%)	89,822 (40.8%)
Estimated gestation at birth (weeks)	39.1 (2.2)	39.0 (2.2)
Multiple births this pregnancy – yes	2575 (1.6%)	3794 (1.7%)

The estimated costs of implementing AFFIRM, RFM visits, and IoLs are reported in Table 2. Following the introduction of the intervention (post-AFFIRM period), the direct costs were £95,126 greater per 1,000 births than for pre-AFFIRM standard care. The increased costs were largely due to RFM attendances (both the number of RFM attendances and the cost per RFM attendance increased due to the increase in number of scans conducted) and IoLs. Based on the number of births at participating sites in the post-AFFIRM trial period (*n* = 228,273), the total additional direct costs during the study were £21.7 m. Although there were more NICU admissions in the post-AFFIRM period, the additional cost per 1,000 births was minimal (£5,792). Intrapartum costs, which incorporate the increased proportion of deliveries by Caesarean-section in the post-AFFIRM period (28.3% versus 25.5% [11] i.e. 2.8% more), were £77,000 higher per 1,000 births in the post-AFFIRM period. The cost-saving associated with the lower stillbirth rate observed post-AFFIRM is minimal, an estimated £369 per 1,000 births.

**Table 2:** Direct and secondary costs associated with the AFFIRM intervention

	**Cost per 1000 births**			
	Pre-AFFIRM	Post-AFFIRM	Difference ^e^	
Direct intervention costs				
Training	£0	£367	£367	
Leaflets	£0	£460	£460	
RFM attendances ^a^	£35,627	£85,511	£49,884	
Inductions of labour	£282,599	£327,012	£44,413	
*Total *^*c*^	*£318,225*	*£413,351*	*£95,126*	
Secondary costs				
NICU admissions > 48 h ^b^	£63,454	£69,246		£5,792
Intrapartum costs ^c^	£2,733,000	£2,810,000		£77,000
Stillbirth costs (per 1,000 births) ^d^	£4,765	£4,396		£- 369

^a^ Pre-AFFIRM RFM attendances (*n* = 261/1000 births) and post-AFFIRM attendances (*n* = 495/1000 births) are from audit data from 11 study sites. Audit data also showed that the proportion of RFM attendances which resulted in an additional ultrasound scan was 30% pre-AFFIRM and 59% post-AFFIRM

^b^ Unit cost for NICU admissions multiplied by observed admission rate for each period (pre-AFFIRM 50.9/1000 births; post-AFFIRM 55.5/1000 births)

^c^ Intrapartum costs do not include the cost for inductions of labour. Only the cost of Caesarean section or vaginal birth are included here

^d^ Unit cost for stillbirths multiplied by observed stillbirth rate for each period (pre-AFFIRM 4.38/1,000 births; post-AFFIRM 4.04/1,000 births)

^e^ Values reported in the table are rounded to nearest whole £

Table 3 summarises the results from the primary and sensitivity trial-based economic evaluations. When the estimated costs of implementing AFFIRM (£95,126 per 1,000 births) are considered alongside the point estimate for the reduction in number of stillbirths observed in AFFIRM (5 per 10,000 births i.e. 0.5 per 1,000 births) the cost per stillbirth avoided is £190,251. The assumption with the greatest impact on the ICER was the impact of AFFIRM on the number of stillbirths avoided. The true ICER is likely to be between £86,478 per stillbirth avoided (if there were 11 fewer stillbirths per 10,000 births) and being dominated (i.e. more costly and less effective – if there were 3 *more* stillbirths per 10,000 births). The other assumption with a big impact on the ICER is the exclusion of costs associated with IoL, reducing the cost per stillbirth avoided to £101,424. However, while it is not possible to say how much of the increase in IoLs is due to AFFIRM, excluding all the increase is an unrealistic underestimation. The same is true about the increase in Caesarean-section deliveries, which when included in the analysis increases the cost per stillbirth avoided to £344,251.

**Table 3:** Base case and one-way sensitivity analyses for trial-based economic evaluation

**BASE CASE**	**per 1000 births**			**Cost/stillbirth avoided**
	**Pre-AFFIRM cost**	**Post-AFFIRM cost**	**Difference**	
	£318,225	£413,351	£95,126	£190,251
90% increase in RFM visits, 59% of post-AFFIRM RFM visits have additional scan, direct costs included (training, leaflets, RFM attendances, IoL) Impact of AFFIRM 5 fewer stillbirths per 10,000 births				
**Sensitivity analyses**				
Assume there is a 110% increase in RFM visits	£314,774	£413,351	£98,577	£197,154
Assume that 30% of post-AFFIRM RFM visits have a scan	£318,225	£395,407	£77,182	£154,364
Assume that 75% of post-AFFIRM RFM visits have a scan	£318,225	£423,251	£105,026	£210,051
Exclude training cost	£318,225	£412,984	£94,759	£189,517
Exclude costs of IoL	£35,627	£86,338	£50,712	£101,424
Include cost of NICU admissions	£381,679	£482,597	£100,918	£201,836
Include increased intrapartum costs (due to Caesarean-sections) post-AFFIRM ^a^	£318,225	£490,351	£172,126	£344,251
Include in-hospital cost savings associated with fewer stillbirths	£318,225	£412,807	£94,582	£189,163
Lower bound of impact of AFFIRM – 3 more stillbirths per 10,000 births	£318,225	£413,351	£95,126	dominated
Upper bound of impact of AFFIRM – 11 fewer stillbirths per 10,000 births	£318,225	£413,351	£95,126	£86,478

^a^ 2.8% more births were via Caesarean-section in the post-AFFIRM period (28.3% versus 25.5%), increasing the intrapartum costs by £77/birth; *RFM* = Reduced fetal movements, *IoL* = Induction of labour, *NICU* = Neonatal intensive care unit

The results of the primary and one-way sensitivity model-based analysis are summarised in Table 4. In the scenario where AFFIRM was implemented across Great Britain, there were an estimated 324 fewer perinatal deaths and a budget impact of a £61,851,400 increase in costs to the NHS. This gives an ICER of £190,899 per death avoided. Ten thousand random pairs of incremental costs and incremental effects (deaths) generated by the model were plotted on an incremental cost-effectiveness plane (shown in Supplementary Material—Figure S2). The points are spread across the four quadrants of the plane, demonstrating uncertainty in the results. The majority of the points (56%) were in the north-east quadrant, indicating higher costs and more live births alive at 7 days postpartum (i.e. fewer perinatal deaths) after the implementation of AFFIRM compared to before. Overall, 74% of the points were in quadrants where there were there more live births with AFFIRM than without. Adding the cost of NICU admissions increased the ICER to £203,319 per death avoided, adding intrapartum costs increased it to £355,594, and adding an additional cost for healthcare resources following a baby death reduced the ICER to £189,812 per death avoided. When the outcome was restricted to stillbirths prevented only, there were an estimated 249 fewer stillbirths following the implementation of AFFIRM, giving an ICER of £248,399 per stillbirth avoided.

**Table 4:** Base case and one-way sensitivity analyses for model-based economic evaluation

	**Pre-AFFIRM total cost [mean and 95% CI]**	**Post-AFFIRM total cost** **[mean and 95% CI]**	**Difference**	**Pre-AFFIRM total deaths [mean rate **^**a**^** and 95% CI]**	**Post-AFFIRM total deaths** **[mean rate **^**a**^** and 95% CI]**	**Difference**	**cost/death avoided**
**BASE CASE – estimated costs and deaths for 693,000 births**	£252,784,813 [£365; £186–611]	£314,636,213 [£454; £258–718]	£61,851,400	3883 [5.6; 4.6–6.8]	3559 [5.1; 4.2–6.1]	324	£190,899
SENSITIVITY ANALYSES							
Include cost of NICU admissions longer than 48 h	£306,784,076 [£443; £255–693]	£372,659,441 [£538; £335–806]	£65,875,365	3883 [5.6; 4.6–6.8]	3559 [5.1; 4.2–6.1]	324	£203,319
Include increased intrapartum costs (due to Caesarean-sections) post-AFFIRM	£2,146,753,667 [£3,098; £2,919–3,344]	£2,261,966,261 [£3,264; £3,068–3,528]	£115,212,594	3883 [5.6; 4.6–6.8]	3559 [5.1; 4.2–6.1]	324	£355,594
Include in-hospital costs associated with deaths	£257,010,231 [£371; £192–617]	£318,509,304 [£460; £264–723]	£61,499,073	3883 [5.6; 4.6–6.8]	3559 [5.1; 4.2–6.1]	324	£189,812
Stillbirths only (not neonatal deaths)	£252,784,813 [£365; £186–611]	£314,636,213 [£454; £258–718]	£61,851,400	2,980 [4.3; 3.6–5.1]	2,731 [3.9; 3.2–4.7]	249	£248,399

^a^ death rate per 1000 births; *NICU* = Neonatal intensive care unit

## Discussion

### Main findings

The AFFIRM intervention was associated with increased costs compared to pre-AFFIRM standard care. The primary drivers of these costs are the increases in RFM attendances (including cost of ultrasound scans) and IoL. The trial-based primary estimate is that for every 1,000 births at the study sites, the AFFIRM intervention was associated with £95,126 higher costs to the NHS than pre-AFFIRM standard care. The primary model-based estimate for implementing the AFFIRM intervention across Great Britain is a cost of £61.9 m for one year. There is uncertainty around the impact of the intervention (the reduction in stillbirths observed in the trial was not statistically significant). There is also likely to be residual confounding in the stepped-wedge design (i.e. between intervention effects and secular trends) so even after adjusting for time it is not possible to fully attribute either the entire increase in costs (e.g. for IoL) or the entire change in the number of baby deaths to the AFFIRM intervention. Therefore, it is appropriate to consider the results as a range of plausible ICERs for the cost per death avoided, from the best-case scenario estimate of £86,478/stillbirth to AFFIRM being “dominated” by standard care (i.e. higher costs and more stillbirths/neonatal deaths).

### Strengths and limitations

A strength of this analysis is that the data were collected across 33 different centres, reflecting the diversity of maternity units in Great Britain, Northern Ireland, and Ireland. The economic analysis used a combination of routinely collected data from electronic healthcare records and audit data. The benefits of using routine data to capture maternity outcomes include the relative ease of collecting a large amount of data (especially important for rare events) and that routine data can be more accurate than self-reported outcomes which may be subject to recall bias and loss to follow-up [19]. A limitation is that routine data from maternity settings may include recognised coding errors in hospital episode statistics [20], but as this would affect both the before and after time periods in this analysis the impact of this should be minimal. The data on NICU admissions was recorded as a binary variable coding whether or not there was an admission, and data on the length of the admissions were not available. The audit data were collected over a one-month period in a sub-group of centres participating in the AFFIRM study and so may not be a true reflection of the resources required across all sites or capture fluctuations over time. In addition, as it was not possible to link individual-level data on RFM attendances to individual-level data on birth outcomes, the costs associated with the increased number of RFM attendances were attributed across the whole sample in the post-AFFIRM period. This means that some of the incremental cost is unrelated to some of the incremental effects. Another limitation is that the analyses (for clinical and cost-effectiveness) did not allow for correlations to decay over time, which is an emerging approach in the analysis of data from stepped wedge trials.

A key strength of this analysis is the use of one-way sensitivity analyses to explore the impact of assumptions on the ICERs, for example the impact of varying the costs derived from audit data. There was considerable overlap between the ICERs across the one-way sensitivity analyses from both the trial and the model. This is reassuring as it suggests minimal impact of the differences between the analyses (described in the Methods section), namely the approach to handling babies coded as both stillbirths and early neonatal deaths and the exclusion of perinatal deaths when the baby was < 24 weeks gestation.

In the economic model, probabilistic sensitivity analysis was used to explore uncertainty in the parameters and plot a cost-effectiveness plane which shows the proportion of results which suggest that the intervention is beneficial (74%). Broadly speaking, the analysis took a Bayesian-type approach whereby the probability of different outcomes is considered, rather than a frequentist approach which focuses on the statistical significance of an outcome and rejection of a null hypothesis. This is particularly useful when investigating infrequent events like perinatal deaths, as it does not rely on statistical power and can generate useful evidence that can inform real-world decision-making.

One limitation of the current analysis is that differences in fidelity of implementation have not been incorporated in the analysis. The implication of this is that it is not possible to determine the relationship between level of implementation and cost-effectiveness of the intervention or explore whether some centres are able to implement the intervention more efficiently than others, without a loss of effect. Although in the main trial analysis, there was little difference in the effectiveness of the AFFIRM care package across the whole sample (OR 0.90) and when the analysis was restricted to maternity units which self-reported that they were adherent to the intervention (OR 0.88) [11], which suggests that refining the intervention to improve adherence may have a minimal impact on effectiveness. There may have been changes in national or local policy aimed at reducing baby deaths that occurred during the AFFIRM study which changed practice in the study sites. Similarly, due to the confounding in the stepped-wedge design between intervention effects and secular trends, it was not possible the isolate the impacts of these changes from the impacts of the AFFIRM intervention.

The timescale for these economic evaluations did not extend beyond the early perinatal period. The longer-term costs (e.g. greater level of care in pregnancy following a perinatal death, increased likelihood of recurrent Caesarean sections) and health impacts (e.g. impact of baby death on parents) are not taken into account. There may be benefits in subsequent pregnancies of having raised awareness of RFM with pregnant women during the study. Similarly, although healthcare professionals complete the training package once, they may have continued their enhanced management of women presenting with RFM beyond the study period.

### Interpretation

The total cost of implementing AFFIRM (over and above standard care) across Great Britain was estimated to be £61,851,400/year. Although this seems like a large amount, in 2014/15 approximately £2.5bn was spent in the NHS on maternity services (with 664,399 births) and in 2019/20 maternity claims handled by the NHS Resolution (formerly NHS Litigation Authority) accounted for £5.7bn (69% of a total £8.3bn annual incurred cost of harm) [21].

There is not a commonly used threshold for cost-effectiveness in terms of cost per baby death avoided and not many published studies which have used this metric. The estimated cost per stillbirth avoided associated with a national roll-out of the Saving Babies Lives Care Bundle (a bundle of care developed by the UK NHS which includes raising awareness of RFM) was estimated to be between £141,312 and £221,690 which overlaps with the estimates for AFFIRM [22]. However, that analysis did not include the cost of RFM attendances or staff time to complete training and the reduction in stillbirths was greater (8 fewer stillbirths per 10,000 births). A paper by Bhutta et al., which was part of the 2011 Lancet Stillbirth Series, reported estimated costs and stillbirths averted if different interventions were available globally [23]. For countries with a ‘low’ stillbirth rate the cost per stillbirth averted ranged from £7,800 for detection and management of gestational hypertension to £203,333 for detection and management of gestational diabetes.

A key area for future research would be to explore and potentially establish meaningful thresholds that decision-makers are willing to pay to prevent a stillbirth. An alternative would be to measure the impact of stillbirths (and neonatal deaths) in a metric already used in decision-making such as the quality adjusted life year (QALY), for which many countries have established willingness to pay thresholds. Similarly, future work should focus on producing a robust and defensible estimate of the impact of stillbirth in terms of the number of QALYs lost. The impact of baby loss on parents and families is well-established and so it is imperative that the perspectives of bereaved parents are incorporated in future research [24].

It is possible that by training healthcare professionals to recognise and act upon RFM, the degree of medical intervention in childbirth may increase inappropriately e.g. “too much intervention, too soon” [25]. This would also drive-up resource use and costs. In one sensitivity analysis reported here, when intrapartum costs were included (to account for an increase in Caesarean-section deliveries in the post-AFFIRM period), the ICER for the cost per perinatal death avoided increased by over 80%. However, it is not possible to identify what proportion of these costs could or should be attributed to the AFFIRM intervention. A recent study in Sweden comparing routine care with an intervention to increase awareness of RFM among pregnant women (but not healthcare professionals) reported a lower likelihood of Caesarean delivery in the intervention group [26]. Although this may be particular to the Swedish context, it highlights the complexity of designing, implementing, and evaluating the impact of ‘care bundle’ interventions.

Elements of the AFFIRM care bundle were designed to be low-cost and low-tech, for example the use of paper leaflets for raising awareness of RFM among pregnant women rather than an electronic or web-based approach. One benefit of this is that the leaflets were equally accessible to pregnant women, regardless of their ability to access the internet. However, as the use of eHealth and telemedicine expands it may be possible to use at-home monitoring (e.g. cardiotocography) in the management of perceived RFM. There are likely to be implications of the use of new technologies for both costs and birth outcomes and so it will be important to fully evaluate their impact.

## Conclusions

In conclusion, there are additional costs associated with providing the AFFIRM intervention and there is uncertainty around the impact of the AFFIRM intervention on the number of perinatal deaths. While there is not a pre-defined cost that decision-makers are willing to pay to avert perinatal deaths, the cost of AFFIRM should be considered alongside both the lifelong health, social, and economic impacts on the families who experience perinatal deaths and the costs of obstetric-related legal claims. In the context of high-income countries with relatively low rates of perinatal deaths, small, incremental reductions of potentially avoidable deaths are the necessary focus of research. However, designing clinical trials to detect and measure these benefits is challenging. Model-based research and use of routinely collected electronic health records are vital tools in unlocking important answers.

## Supplementary Information

**Additional file 1 **

## Abbreviations

CI: Confidence interval
ICER: Incremental cost-effectiveness ratio
IoL: Induction of labour
NHS: National Health Service
NICU: Neonatal intensive care unit
NRS: National Records of Scotland
ONS: Office for National Statistics
QALY: Quality adjusted life year
RFM: Reduced fetal movements
UK: United Kingdom
