# Supplementary material for: Awareness of fetal movements and care package to reduce fetal mortality (AFFIRM): a trial-based and model-based cost-effectiveness analysis from a stepped wedge, cluster-randomised trial
Source: BMC Pregnancy Childbirth. 2022 Mar 22;22:235. doi: 10.1186/s12884-022-04563-9 (PMC8941740; doi:10.1186/s12884-022-04563-9)
Supplement: Supplementary file 1 — Additional file 1 [file 12884_2022_4563_MOESM1_ESM.docx]

**Supplementary Material**

**Awareness of fetal movements and care package to reduce fetal mortality (AFFIRM): a trial-based and model-based cost-effectiveness analysis from a stepped wedge, cluster-randomised trial**

Elizabeth M Camacho, Sonia Whyte, Sarah J Stock, Christopher J Weir, Jane E Norman, Alexander EP Heazell

*Additional description of the methods used to estimate training costs*

A total of 2317 people completed the AFFIRM online training (79% midwives, 4% consultants, 4% SHO/registrar, 13% other). The training took 45 minutes to complete. The unit cost-per-hour for the different staff roles of people who completed the training was multiplied by the number of people and duration of training to estimate a total cost. The estimated total training cost was £83,822. This cost was divided by the number of births during the post-implementation period of the trial (n= 228,273) to estimate a training cost per 1000 births (£367). This cost may be an overestimation as it is unknown whether refresher training would be required to continue delivering the intervention over a longer time period i.e. if training is one-off then the training cost per birth would be lower. However, it may also be an underestimation as the staff turnover is not accounted for in this estimate. These uncertainties will be explored in sensitivity analyses.

**Table S1.** Unit costs used to estimate costs associated with the AFFIRM intervention

| Resource | Unit cost | Source |
| --- | --- | --- |
| Direct costs | | |
| Training | NHS band 6 £35  Consultants £82  FY1/FY2 £23  Unclassified £37^a^ | Study logs recorded 2317 individuals in various roles completed training, lasting 45 minutes (unit cost is per 45 minutes) (1). |
| Leaflets | £0.46 | Amount invoiced by supplier of leaflets, inflated to 2019 using health cost inflation indices (1). |
| RFM attendances | £99 attendance  £125 ultrasound scan | NHS unit costs for outpatient midwifery visit (all RFM attendances) and cost for non-routine ultrasound scan (a proportion of RFM visits^b^) (2). |
| Induction of labour | £894 | NHS unit costs for induction of labour (2). |
| Secondary costs | | |
| NICU admissions >48 hours | £1,247 | Weighted mean unit cost of high dependency and intensive care (neonatal critical care) admissions (2). |
| Intrapartum costs | Vaginal £2,046  Caesarean section £4,746 | Weighted mean NHS unit costs for vaginal and Caesarean section births (2) and proportion of Caesarean sections used to calculate weighted mean intrapartum cost for pre- and post-AFFIRM ^c^ |
| In-hospital costs following stillbirth | £1088 | Inflated to 2018/19 from Campbell et al. (3) |

^a^ Where the role stated by the person completing the training did not map onto a specific NHS band, the weighted mean unit cost of the identified roles (£37) was used.

^b^ pre-AFFIRM 30%; post-AFFIRM 59% of RFM visits resulted in a non-routine ultrasound scan

^c^ pre-AFFIRM 34% Caesarean section (average intrapartum cost £2,733); post-AFFIRM 39% Caesarean section (average intrapartum cost £2,810)

**Figure S1.** Model structure for the post-AFFIRM period; the structure is replicated for the pre-AFFIRM period.


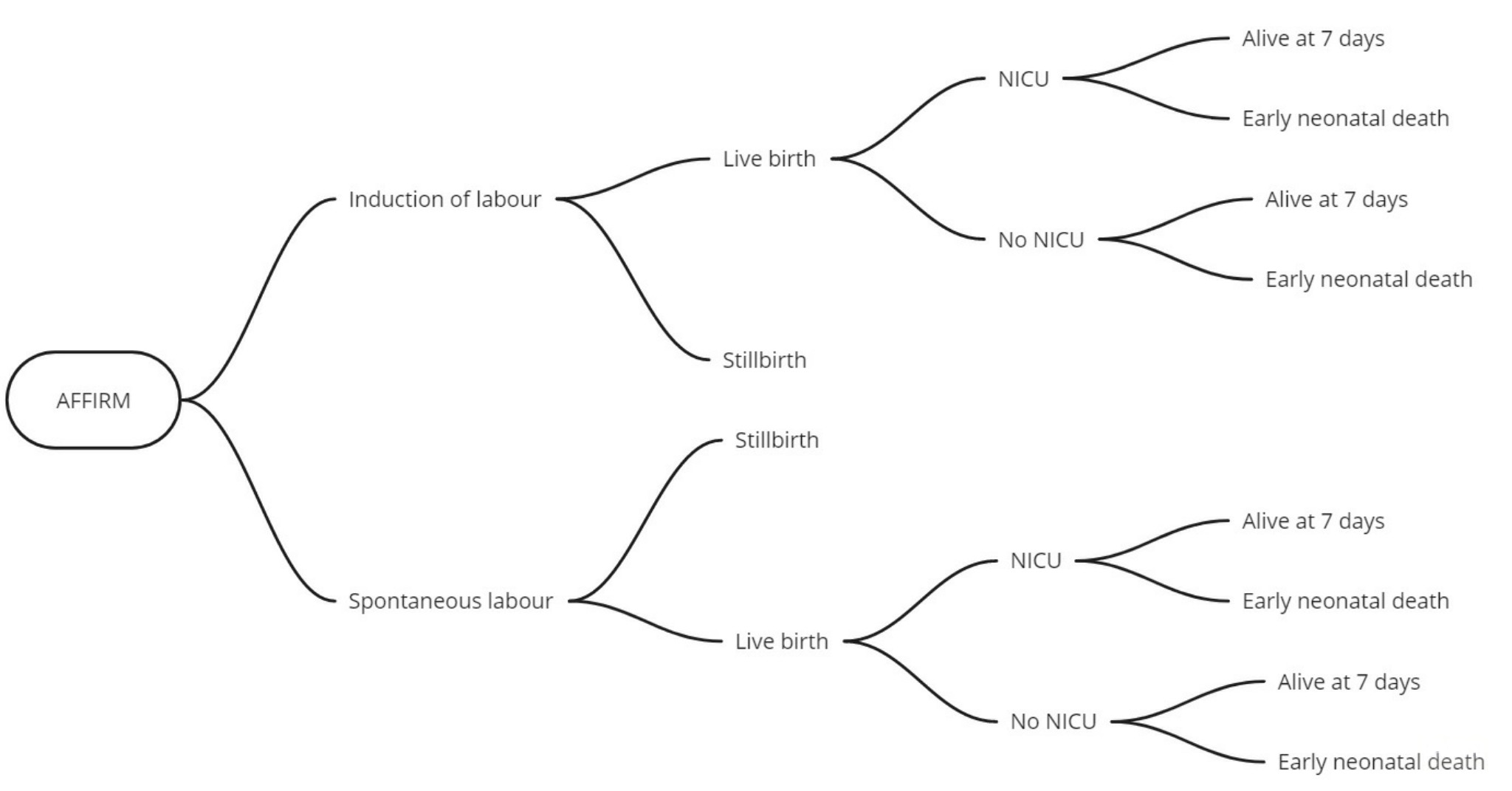


**Table S2.** Proportion of births following different pathways through the decision tree, before and after implementation of the intervention

|  | **Sub-group** | **Event** | **Probability (SD)** |
| --- | --- | --- | --- |
| **Before AFFIRM** | All births | IoL  Natural onset | 0.3680 (0.096) |
|  |  |  | 1-AOP |
|  | Induced labour | Live birth  Stillbirth | 0.9957 (0.005) |
|  |  |  | 1-AOP |
|  | Induced labour + live birth | No NICU  NICU | 0.9378 (0.022) |
|  |  |  | 1-AOP |
|  | Induced labour + live birth + no NICU | Alive at 7 days  Early neonatal death | 0.9987 [0.006] |
|  |  |  | 1-AOP |
|  | Induced labour + live birth + NICU | Alive at 7 days  Early neonatal death | 0.9985 [0.007] |
|  |  |  | 1-AOP |
|  | Spontaneous labour | Live birth  Stillbirth | 0.9957 (0.005) |
|  |  |  | 1-AOP |
|  | Spontaneous labour + live birth | No NICU  NICU | 0.9369 (0.023) |
|  |  |  | 1-AOP |
|  | Spontaneous labour + live birth + no NICU | Alive at 7 days  Early neonatal death | 0.9987 [0.006] |
|  |  |  | 1-AOP |
|  | Spontaneous labour + live birth + NICU | Alive at 7 days  Early neonatal death | 0.9986 [0.007] |
|  |  |  | 1-AOP |
| **After AFFIRM** | All births | IoL  Natural onset | 0.4118 (0.099) |
|  |  |  | 1-AOP |
|  | Induced labour | Live birth  Stillbirth | 0.9960 (0.006) |
|  |  |  | 1-AOP |
|  | Induced labour + live birth | No NICU  NICU | 0.9328 (0.019) |
|  |  |  | 1-AOP |
|  | Induced labour + live birth + no NICU | Alive at 7 days  Early neonatal death | 0.9988 [0.004] |
|  |  |  | 1-AOP |
|  | Induced labour + live birth + NICU | Alive at 7 days  Early neonatal death | 0.9988 [0.004] |
|  |  |  | 1-AOP |
|  | Spontaneous labour | Live birth  Stillbirth | 0.9961 (0.005) |
|  |  |  | 1-AOP |
|  | Spontaneous labour + live birth | No NICU  NICU | 0.9324 (0.021) |
|  |  |  | 1-AOP |
|  | Spontaneous labour + live birth + no NICU | Alive at 7 days  Early neonatal death | 0.9988 [0.004] |
|  |  |  | 1-AOP |
|  | Spontaneous labour + live birth + NICU | Alive at 7 days  Early neonatal death | 0.9988 [0.005] |
|  |  |  | 1-AOP |

AOP = all other probabilities; cells denoted as 1-AOP will be derived as the complimentary probability of the other branches from the same node

**Figure S2.** Cost-effectiveness plane showing distribution of 10,000 pairs of costs and effects (perinatal deaths) derived from the model

**
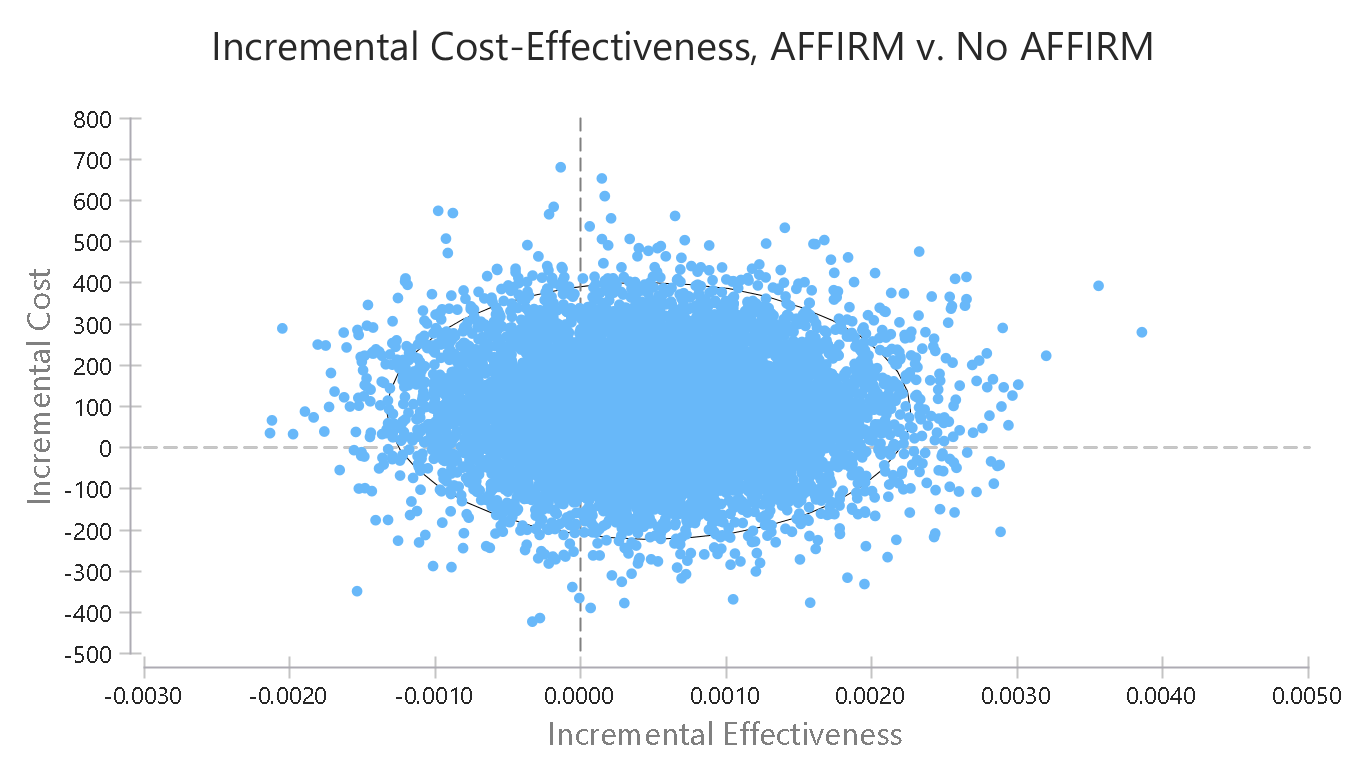
**

REFERENCES

1. Curtis LA, Burns A. PSSRU: Unit Costs of Health and Social Care 2019. Kent: PSSRU; 2019.

2. NHS England. National Schedule of Reference Costs 2018/19 [Internet]. NHS England; 2019. Available from: https://www.england.nhs.uk/national-cost-collection/#ncc1819

3. Campbell HE, Kurinczuk JJ, Heazell AEP, Leal J, Rivero-Arias O. Healthcare and wider societal implications of stillbirth: a population-based cost-of-illness study. BJOG Int J Obstet Gynaecol. 2018;125(2):108–17.
